# Sanatorium Complaints

**Published:** 1919-12-13

**Authors:** 


### Sanatorium Complaints.

At a recent meeting of the Yorkshire West Riding Insurance Committee report was made of an investigation into complaints laid against the Mitchell's Memorial Home, in use for tuberculous soldiers. One feature of the management of this institution which was condemned was that the lady in charge was aided by an official known apparently as the "sergeant-major," who supervised such matters as exercise. It was pointed out that after serving in the Army these men would probably have had enough already of such an official, and that to find him again at the sanatorium would disgust them considerably. The Chairman of the Committee spoke of men stopping out at night drinking in public-houses, and so forth. The institution in question had, however, according to the newspaper report before us, been organised in a hurry to meet the undoubtedly very great demand for accommodation. We remember an instance further south where an ex-Army N.C.O. was installed as steward some years ago and had to be removed after a great number of resident medical officers had resigned in protest against his activities. In short, it is the old tale. Many people try to modify and improve upon the plan of Walther and Brehmer, and, indeed, Bodington, whereby the resident medical man holds an authoritative position. All those who do fail—and the result of their many failures is to make British public sanatoriums a travesty of what should be—and to get these places a bad name amongst the working class.

